# Improving control of the Mpox outbreak: a national cross-sectional study on the knowledge, attitudes, and influencing factors among frontline healthcare professionals in Ethiopia

**DOI:** 10.3389/fpubh.2025.1551163

**Published:** 2025-07-02

**Authors:** Getahun Fetensa, Bizuneh Wakuma, Merga Besho, Girma Yadesa, Jilcha Gugsa, Derara Girma Tufa, Feyiso Bati, Kitesa Biresa Duftu, Tadesse Tolossa

**Affiliations:** ^1^Department of Nursing, Institute of Health Sciences, Wollega University, Nekemte, Ethiopia; ^2^Department of Health, Behavior, and Society, Institute of Health, Jimma University, Jimma, Ethiopia; ^3^Center for Evidence Synthesis, Support and Development in Africa (CESDA), PLC, Addis Aba, Ethiopia; ^4^Department of Nursing, School of Health Science, Ambo University Woliso Campus, Woliso, Ethiopia; ^5^Department of Midwifery, Institute of Health Sciences, Wollega University, Nekemte, Ethiopia; ^6^Department of Nursing, College of Medical and Health Sciences, Dire Dawa University, Dire Dawa, Ethiopia; ^7^Oromia Physician Association, Addis Ababa, Ethiopia; ^8^Public Health Department, College of Health Sciences, Salale University, Fiche, Ethiopia; ^9^Department of Public Health, College of Medicine and Health Sciences, Dire Dawa University, Dire Dawa, Ethiopia; ^10^Department of Epidemiology, Faculty of Public Health, Institute of Health, Jimma University, Jimma, Ethiopia; ^11^Department of Public Health, Institutes of Health Sciences, Wollega University, Nekemte, Ethiopia; ^12^Deakin Health Economics, School of Health and Social Development, Institute for Health Transformation, Deakin University, Geelong, VIC, Australia

**Keywords:** Mpox, knowledge, attitude, health professionals, Ethiopia

## Abstract

**Introduction:**

Monkeypox (Mpox) has emerged as a global public health concern, with ongoing outbreaks in non-endemic countries affecting various aspects of the healthcare system. This study aimed to determine the knowledge, attitudes, and factors associated with Mpox among healthcare professionals in Ethiopia.

**Methods:**

A national cross-sectional study was conducted in Ethiopia from 31 August 2024 to 10 September 2024, involving 749 frontline healthcare professionals. Data were collected via an online survey using Google Forms, with questionnaires distributed through widely used social media platforms such as Email, Telegram, and WhatsApp. Participants were recruited using a snowball sampling technique to ensure diverse representation among frontline health workers.

**Result:**

A total of 749 healthcare professionals participated in the study, yielding a response rate of 93.6%. More than half (56.5%) of the participants demonstrated good knowledge about Mpox, while 51.5% showed a positive attitude toward Mpox. Statistically significant factors associated with better knowledge of healthcare professionals toward Mpox included being male [adjusted odds ratio (AOR) = 1.61], being in the 25–30 years age group (AOR = 2.29), and having a history of coronavirus disease 2019 (COVID-19) vaccination (AOR = 1.84). Factors significantly associated with a positive attitude toward Mpox included having good knowledge (AOR = 1.41), being male (AOR = 2.07), holding a diploma (AOR = 1.96), earning a monthly income between 8,018 and 9,057 ETB (AOR = 2.83), and identifying as an Orthodox Christian (AOR = 1.65).

**Conclusion:**

Knowledge and attitude toward Mpox and its prevention among healthcare professionals in Ethiopia are found to be suboptimal. Significant efforts are needed to control and prevent outbreaks in Ethiopia by enhancing the capability of healthcare professionals. Factors such as a history of COVID-19 vaccination, male sex, and being in the 25–30 age group were significantly associated with knowledge of the disease and its prevention. Moreover, factors such as male sex, a diploma-level education, monthly income, and being an Orthodox religion follower were linked to positive attitudes toward Mpox and its prevention. Further studies are needed to tackle the perceived challenges of controlling the outbreak among potential stakeholders, including healthcare professionals working in rural areas, to support the current findings.

## Introduction

Monkeypox (Mpox) is a zoonotic infection caused by the Mpox virus, a double-stranded DNA virus of the genus *Orthopoxvirus* within the *Poxviridae* family (1, 2). The virus is divided into two genetically distinct clades, Clade I (formerly the Congo Basin clade) and Clade II (formerly the West African clade). Clade I is more virulent, with a case fatality rate of approximately 10% in African outbreaks. Clade II includes two subclades, IIa and IIb. Clade IIb is responsible for the current global outbreak, while new cases of Clade IIa continue to be reported (3). Mpox can also spread from human to human through contact with bodily fluids, skin lesions, internal mucosal surfaces, such as in the mouth or throat, respiratory droplets, and contaminated objects (4).

The ongoing outbreak in non-endemic countries has made the Mpox disease a global concern (5), leading to its declaration as a global public health emergency by the World Health Organization (WHO) on 23 July 2022 (6). Since 1 January 2022, the WHO has received reports of Mpox cases from 121 Member States in all 6 WHO regions. As of 31 July 2024, 103,048 laboratory-confirmed cases and 186 probable cases, including 229 deaths, had been reported to the WHO. Particularly, as of 1 September 2024, 6,303 laboratory-confirmed cases, including 54 deaths, had been reported to the WHO from African countries (7). The Democratic Republic of Congo (DRC) has recorded the most Mpox cases among the 13 African countries, with a total of 19,667 infections and 575 deaths (8).

Real-time polymerase chain reaction (PCR) is currently used as the reference molecular technique to diagnose Mpox in patients (2). Similarly, the prevention of Mpox requires a two-pronged strategy. The initial priority should be to treat sick people and provide post-exposure care. The next step is to prioritize diagnostic tests and vaccine availability at the public health and policy/administrative levels (9). Furthermore, optimizing knowledge regarding Mpox should be prioritized to highlight the necessity of preventive measures, such as vaccination, to protect the public (10). Subsequently, to ensure optimal preparedness and prompt responses to Mpox, capacity-building initiatives for frontline healthcare workers (HCWs) are suggested (11).

According to analyses, the immigration crisis in the Canary Islands and the Mpox virus are linked to the Mpox outbreak (12). This illness, which largely affects Africa’s tropical rainforests, has primarily targeted the most vulnerable and impoverished people (13). Communities residing in rural areas, small towns with less than a thousand residents, humid evergreen tropical forests, and areas close to the human–animal interface have been affected by these outbreaks (14). International travel, migration, climate change, and global interconnectedness all contribute significantly to the disease’s recurrence (15). Being HIV-positive, coming into contact with bush meat or rodents, and not having received a smallpox vaccination were all found to be risk factors for the Mpox outbreak (16).

Mpox has a broad impact on many other domains, including negative effects on healthcare, social, psychological, and economic spheres. Self-isolation, quarantine, and social isolation are the results of the outbreak. Consequently, mental health problems such as sadness, anger, frustration, depression, and anxiety become imminent (17). During the Mpox outbreak of 2017–2018, there were many concerns regarding the social isolation and stigmatization of patients, survivors, and their families (18).

In 2022, three strategic objectives were established by the WHO operational planning guideline to contain the Mpox outbreak, which included interrupting human-to-human transmissions, decreasing zoonotic transmission, and safeguarding vulnerable populations. Furthermore, the recently released and previous WHO operational planning guidelines have stressed the urgency to step up research efforts to close important information gaps in the areas of Mpox epidemiology, transmission dynamics, clinical presentation, and the efficacy of therapies. The rate at which the virus is spreading, continuous surveillance with robust laboratory investigation, sustainable risk communication and community engagement (RCCE), ensuring vaccination of the population at risk, and understanding the knowledge and attitudes toward Mpox are important in the prevention and control of the disease (6, 19–22).

Kenya reported the first confirmed Mpox case in July 2024, which implies that the disease is quickly spreading between countries (22). As Ethiopia shares a significant dry port and airport boundary with Kenya, it is concerning to note that these outbreaks can easily enter Ethiopia. This fact necessitates a nationwide assessment of knowledge and attitudes toward Mpox and its prevention measures among healthcare professionals in Ethiopia.

Our investigation has revealed the research gaps, which are detailed below. First, knowledge generation, research, and innovation are critical pillars in the WHO’s past and current operational guidelines, as well as the interim guidance on the Mpox strategic preparedness and response plan, respectively. Second, the WHO declared the outbreak a Public Health Emergency of International Concern (PHEIC), and the Africa Centres for Disease Control and Prevention (Africa CDC) declared it a Public Health Emergency of Continental Security (PHECS) (6, 22).

On the other hand, Kenya—sharing a high mobile border with Ethiopia—recently reported its first confirmed Mpox case. In light of this, we realized that assessing the nationwide knowledge and attitudes of frontline healthcare professionals toward Mpox would provide significant input for emergency preparedness and response.

## Method

### Study setting

This study was conducted in Ethiopia, a country located in the Horn of East Africa. As the second most populous nation on the continent, Ethiopia reported an estimated population of 123 million in 2020. In 2021, approximately 181,872 healthcare professionals were employed in public health facilities across the country.

### Study design and study period

This online cross-sectional study was conducted among healthcare professionals in Ethiopia from 31 August 2024 to 10 September 2024.

### Study population

Our study population included frontline healthcare professionals, such as doctors, nurses, midwives, laboratory technicians, and pharmacists, who are actively involved in diagnosing and treating patients. Administrative staff were not included in the online survey.

### Sample size and sampling techniques

The sample size was determined using a single population proportion formula with the following assumptions: a prevalence of good knowledge from a previous study conducted in Ethiopia (*p* = 38.5) (23), a 5% margin of error, and a 95% confidence level. Accordingly, the calculated sample size was 370. After adding a 10% allowance for a non-response rate and a design effect of 2, the final sample size was 800 healthcare professionals.

The study was conducted using an online survey to gather data from frontline healthcare professionals. The survey tool was carefully designed using online Google Forms, which was chosen for its ease of distribution, user-friendly interface, and ability to efficiently collect and organize responses. The questionnaires were distributed via targeted social media platforms where healthcare professionals are actively engaged, including Email, Telegram, and WhatsApp. A purposive snowball sampling technique was utilized to reach a broader and more diverse group of frontline healthcare professionals. The process involved sending the survey link to an initial group of healthcare professionals. These individuals were selected based on their availability and known connections within the healthcare community. After the initial distribution, the healthcare professionals were encouraged to share the survey link with other healthcare professionals in their networks. This method was particularly effective in reaching healthcare professionals working in diverse settings, including those in remote or hard-to-reach areas. The exclusion criteria were applied only to retired healthcare professionals and those working in a business outside of healthcare services.

### Variables and their measurement

This study explores multiple outcome variables. The primary outcome variable is knowledge of Mpox and its prevention using 23 yes/no questions. Correct answers receive “1” point, while incorrect answers receive “0” points, with total scores ranging from 0 to 23. Scores equal to or greater than the mean were categorized as “good knowledge,” while scores less than the mean were categorized as “poor knowledge” on Mpox and its prevention. The second outcome variable is attitudes toward Mpox and its prevention through 10 items on a 3-point Likert scale (agree, neutral, and disagree). Scores equal to or greater than the mean are classified as “positive attitude,” while those less than the mean are deemed as “negative attitude.” Different explanatory variables were also collected, such as age, residence, type of health facility, experience in years, marital status, educational status, religion, profession, monthly income, role of healthcare professionals, and travel history.

### Data collection tool and its procedure

A survey was developed by reviewing previously published articles, focusing on socio-demographic data, knowledge, and attitudes toward Mpox and its prevention. The questionnaire was prepared in English using Google Forms and distributed through an online platform to healthcare professionals active on social media. The content of the questionnaire was thoroughly reviewed by all authors of the study before being shared with participants. To ensure clarity, the questionnaire was pre-tested on 5% of the participants. Following the pre-test, the investigators discussed the results (ambiguity of the questions) and made the required amendments. Once the final version was agreed upon, the authors shared the responsibilities and began distributing the survey questionnaire. An information sheet was included with the survey questionnaire to clarify its purpose to the participants. The healthcare professionals were also encouraged to share the survey link with their colleagues. Additionally, the initial participants were asked to monitor the progress of data collection within their networks. To reduce the non-response rate, the participants were requested to submit a screenshot of their completed responses. The authors promptly checked the submissions for completeness, accuracy, clarity, and consistency, making corrections as needed to ensure data quality throughout the data collection process.

#### Data quality assurance

The data collection tool was pre-tested on 5% (40 individuals) of the total sample size to ensure it is clearly articulated. Moreover, each question was entered into the Google Form in a must-enter order, unless otherwise indicated. Furthermore, we had restricted multiple responses, with one participant being able to send only a single response. All data were cleaned before the actual data analysis.

### Data analysis and interpretation

Data from the Google Form were downloaded in Excel format from an online data collection form and exported to SPSS version 28 for further analysis and interpretation. Descriptive statistics were performed to explain socio-demographic variables and reported with frequency and percentage. Logistic regression was conducted to assess the relationship between the outcome variables and explanatory variables. Cross-tabulation of both knowledge and attitude was undertaken to check whether the variables within the study fulfilled the logistic regression model assumption, A bivariable analysis was performed for each independent variable separately at a significant level of *p*-value <0.25, and all variables fulfilling the eligibility criteria were included within the final model built independently for knowledge and attitude of participants. Statistical significance was declared at a *p*-value of <0.05. The results were presented using texts and tables.

## Operational definition

### Knowledge of healthcare professionals toward Mpox and its prevention

Healthcare professionals whose total score was greater than the mean score for the knowledge-related questions were categorized as having good knowledge of Mpox and its prevention; those with scores below or equal to the mean were categorized as having poor knowledge.

### Attitude of healthcare professionals toward Mpox and its prevention

The healthcare professionals whose total score was greater than the mean score for the attitude-related questions were categorized as having a positive attitude toward Mpox and its prevention; those with scores below or equal to the mean were categorized as having a negative attitude.

## Result

### Socio-demographic variables

A total of 749 healthcare professionals participated in the study, with a response rate of 93.6%. Approximately half of the respondents (370, 49.4%) were in the age range of 31–34 years. Most of them (637, 85%) and approximately half of them (352, 47.0%) were male and degree holders, respectively. Of the total study participants, 459 (61.3%) were married, 52 (6.9%) had a recent travel history to a country with Mpox outbreak, 173 (23.1%) had a history of COVID-19 infection, and approximately three-fourths of them (72.5%) had taken COVID-19 vaccines (Table 1).

**Table 1:** Sociodemographic characteristics of healthcare professionals in Ethiopia, 2024.

Variables	Categories	Frequency	Percent
Sex of respondents	Male	637	85.0
	Female	112	15.0
Age category in years	19–24	39	5.2
	25–30	198	26.4
	31–34	370	49.4
	35–39	99	13.2
	> + 40	43	5.7
Marital status	Single	283	37.8
	Married	459	61.3
	Divorced	4	0.5
	Widowed	3	0.4
Educational status	Diploma	53	7.1
	Degree	352	47.0
	Master’s and above	344	45.9
Monthly income in ETB	600–6,193	125	16.7
	6,194–8,017	211	28.2
	8,018–9,057	119	15.9
	9,058–15,390	236	31.5
	> = 15,391	58	7.7
Religion	Muslim	94	12.6
	Orthodox	261	34.8
	Protestant	339	45.3
	Catholic	4	0.5
	Other^*^	51	6.8
Work Experience in years	<5 years	258	34.4
	> = 5 years	491	65.6
Residence	Urban	674	90.0
	Rural	75	10.0
Travel history to a Mpox outbreak country	Yes	52	6.9
	No	697	93.1
History of COVID-19 infection	Yes	173	23.1
	No	576	76.9
Contact history with COVID-19-diagnosed people	Yes	360	48.1
	No	389	51.9
Took COVID-19 vaccine	Yes	543	72.5
	No	206	27.5
Known chronic disease	Yes	72	9.6
	No	677	90.4
Have access to information about Mpox?	Yes	713	95.2
	No	36	4.8
Source of information	Social media	371	49.5
	Television and radio	378	50.5
	Colleagues and Medical books	193	25.8

Other*, Adventist, Wakefata, apostolic, Jehovah’s Witness; ETB, Ethiopian Birr.

### Knowledge

Knowledge scores were categorized into low and high. We used the mean score of 12.76 as a cutoff point. A mean score greater than or equal to 12.76 was considered “good knowledge,” while a score less than 12.76 was considered “poor knowledge.” Out of 749 respondents, 423 (56.5%) had good knowledge about Mpox.

The majority of participants (84.2%) correctly understood that there were no confirmed Mpox cases in Ethiopia at the time of this study. Participants responded to how Mpox spreads, with 722 (96.4%) understanding that it is easily transmitted from person to person. Additionally, 667 (89.1%) participants knew Mpox can be transmitted through skin-to-skin contact, and 588 (78.5%) were aware of respiratory droplets as a transmission mode. A total of 669 (89.3%) healthcare professionals identified flu-like symptoms as early signs, and 705 (94.1%) were aware that rashes on the skin are a common symptom. Regarding preventive practices, 627 (83.7%) participants accepted the importance of using hand sanitizers and face masks for Mpox prevention. More than half (425, 56.7%) were aware that individuals vaccinated against smallpox have some immunity against Mpox. Moreover, 462 (61.7%) were aware that there is a specific vaccine for Mpox (Table 2).

**Table 2:** Knowledge of Mpox and its prevention among healthcare professionals in Ethiopia, 2024.

Variables	Response	Frequency	Percent
Mpox is prevalent in West and Central Africa	Yes	52	6.9
	No	697	93.1
There is/are confirmed human Mpox cases in Ethiopia	Yes	118	15.8
	No	631	84.2
Mpox is a viral infection	Yes	723	96.5
	No	26	3.5
Mpox is a bacterial infection	Yes	50	6.7
	No	699	93.3
Mpox is easily transmitted from person to person	Yes	722	96.4
	No	27	3.6
Mpox is transmitted by the bite of an infected animal	Yes	444	59.3
	No	305	40.7
Mpox is transmitted through skin-to-skin contact	Yes	667	89.1
	No	82	10.9
Mpox is transmitted through respiratory droplets	Yes	588	78.5
	No	161	21.5
Mpox can be transmitted by eating insufficiently cooked meat from an infected animal	Yes	489	65.3
	No	260	34.7
International travel is the main source of imported cases of Mpox	Yes	690	92.1
	No	59	7.9
Mpox and smallpox have almost similar signs and symptoms	Yes	597	79.7
	No	152	20.3
Flu-like symptoms (fever, chills, cough, runny nose, fatigue, headache, muscle aches, and backache) are the early signs or symptoms of Mpox	Yes	669	89.3
	No	80	10.7
Rashes on the skin are one of the signs or symptoms of Mpox	Yes	705	94.1
	No	44	5.9
Vesicles on the skin are one of the signs or symptoms of human Mpox	Yes	687	91.7
	No	62	8.3
Diarrhea is one of the signs or symptoms of human Mpox	Yes	343	45.8
	No	406	54.2
Lymphadenopathy (swollen lymph nodes) is one clinical sign or symptom that could be used to differentiate between Mpox and smallpox cases	Yes	595	79.4
	No	154	20.6
Use of hand sanitizers and face masks is important in preventing Mpox	Yes	627	83.7
	No	122	16.3
One management option for symptomatic Mpox patients is to use paracetamol	Yes	594	79.3
	No	155	20.7
Antiviral drugs are required in the management of human Mpox patients	Yes	540	72.1
	No	209	27.9
Antibiotics are required in the management of human Mpox patients	1. Yes	379	50.6
	2. No	370	49.4
People who got the smallpox vaccine are immunized against Mpox	Yes	425	56.7
	No	324	43.3
There is a specific vaccine for Mpox	Yes	462	61.7
	No	287	38.3
There is a specific treatment for Mpox	Yes	232	31.0
	No	517	69.0

### Attitude toward Mpox and its prevention

As per the current study, most participants (708, 94.5%) have an agreed-upon attitude toward having more information about Mpox. Regarding learning about the epidemiology of emerging diseases in different areas, a majority of the participants (711, 94.9%) agreed. Moreover, 577 (77%) participants believed that a restriction on travel to countries with Mpox is important for Mpox control. A total of 579 (77.3%) participants agreed to take the Mpox vaccine if it were available within the country (Table 3).

**Table 3:** Attitude of healthcare professionals toward Mpox and its prevention in Ethiopia, 2024.

Items	Disagree	Neither agree nor disagree	Agree
I am interested in learning more about Mpox disease	24 (3.2%)	17 (2.3%)	708 (94.5%)
I am interested in learning more about the epidemiology of new emerging diseases	19 (2.5%)	19 (2.5%)	711(94.9%)
I think that the Mpox disease can be transmitted to my country	75(10.0%)	110 (14.7%)	564 (75.3%)
I have a bad feeling that the Mpox virus could become a worldwide pandemic	84 (11.2%)	156 (20.8%)	509 (68.0%)
I think that Mpox can add a new burden on the healthcare system of affected countries	31 (4.1%)	53 (7.1%)	665 (88.8%)
I think traveling to Mpox disease-infected countries is risky and should be restricted	67 (8.9%)	105 (14.0%)	577 (77.0%)
I have reservations/doubts about the virus’s genesis as it is now explained	145 (19.4%)	254 (33.9%)	350 (46.7%)
The spread of viruses is a deliberate attempt to reduce the size of the global population	277 (37.0%)	176 (23.5%)	296 (39.5%)
I think Mpox will finally be successfully controlled globally	64 (8.5%)	148 (19.8%)	537 (71.7%)
I will receive the Mpox vaccine if made available	68 (9.1%)	102 (13.6%)	579 (77.3%)

The attitude part included 10 items, and the response to each item was measured on a 3-point Likert scale. A score of 1 represented “Agree,” and 0 represented “Undecided” or “Disagree” (23, 24). The total score ranges from 0 to 10. The mean score of participants’ responses was 7.33, and responses with scores greater than the mean score were categorized as having a positive attitude toward prevention of Mpox, while responses with scores less than 7.33 were categorized as having a negative attitude. Accordingly, 386 individuals (51.5%) demonstrated positive attitudes toward Mpox and its prevention, while 363 individuals (48.5%) showed negative attitudes.

### Factors associated with knowledge of Mpox infection among frontline healthcare professionals

Those variables whose *p*-value was less than 0.25 in bi-variable analysis, such as age, sex, marital status, monthly income, educational level, work experience, and history of COVID-19 vaccination, were included in multivariable logistic regression for further analysis. The multi-variable analysis revealed that age, sex, and history of COVID-19 vaccination were significantly associated with knowledge of Mpox and its prevention.

Male healthcare professionals had 1.6 times (AOR = 1.61, 95% CI = 1.01, 2.56) higher odds of good knowledge compared to their counterparts. The odds of having good knowledge about Mpox infection among healthcare professionals in the 25–30 years age group were approximately two times more likely than those in the age group of 15–24 years (AOR = 2.29, 95% CI = 1.07, 4.87). The knowledge grades regarding Mpox and its prevention among participants increased significantly (AOR = 1.84, 95% CI = 1.31, 2.58) with them having a history of COVID-19 vaccination (Table 4).

**Table 4:** Multivariable logistic regression model for knowledge of Mpox and its prevention and associated factors among frontline healthcare professionals in Ethiopia, 2024.

Variables	Categories	COR	AOR	*p*-value
Age category in years	15–24	Ref	Ref	
	25–30	3.10 (1.54, 6.25)	2.29 (1.07, 4.87)	0.031*
	31–34	2.10 (1.03, 3.99)	1.41 (0.62, 3.16)	0.403
	35–40	2.10 (0.98, 4.45)	1.39 (0.56, 3.45)	0.476
	>40	1.44 (0.60, 3.48)	0.83 (0.30, 2.32)	0.730
Sex of participants	Female	Ref	Ref	
	Male	1.71 (1.14, 2.57)	1.61 (1.01, 2.56)	0.042*
Educational status	Diploma	Ref	ref	
	Degree	1.86 (1.02, 3.40)	1.50 (0.74, 3.02)	0.251
	Master’s	1.87 (1.02, 3.41)	1.56 (0.75, 3.26)	0.232
Monthly income in ETB	600–6,193	Ref	ref	
	6,194–8,017	1.01 (0.65, 1.57)	0.78 (0.47, 1.29)	0.339
	8,018–9,057	1.10 (0.66, 1.84)	0.85 (0.48, 1.53)	0.609
	9,058–15,390	1.08 (0.70, 1.66)	1.00 (0.58, 1.71)	0.987
	>15,390	1.59 (0.90, 2.83)	1.20 (0.61, 2.34)	0.124
Work experience	1–4 years	Ref	ref	
	>4 years	1.02 (0.75, 1.38)	1.11 (0.73, 1.68)	0.623
Marital status	Single	Ref	ref	
	Married	0.79 (0.58, 1.07)	0.81 (0.56, 1.18)	0.288
	Others	0.49 (0.10, 2.26)	0.63 (0.12, 3.18)	0.582
COVID-19 vaccination history	No	Ref	Ref	
	Yes	1.78 (1.29, 2.45)	1.84 (1.31, 2.58)	<0.001*

COR, crude odds ratio; AOR, adjusted odds ratio; ref, reference category; ETB, Ethiopian Birr. *Denotes a significant *p*-value < 0.05.

### Factors associated with attitude toward Mpox and its prevention among frontline healthcare professionals in Ethiopia

In bivariate logistic regression, the variables associated with a good attitude toward Mpox infection were the age of participants, sex of participants, educational status, monthly income, religion, residence, COVID-19 vaccination history, and knowledge about Mpox infection.

Healthcare professionals who had good knowledge about Mpox infection were 1.4 times (AOR = 1.41, 95% CI = 1.02, 1.91) more likely to have a positive attitude toward Mpox prevention. Compared to females, male healthcare professionals were two times (AO = 2.07, 95% CI = 1.30, 3.29) more likely to have a positive attitude toward Mpox prevention. The attitude level of the healthcare professionals also differed significantly across educational levels, with those with a diploma being approximately two times (AO = 1.96, 95% CI = 1.04, 3.68) more likely to have a positive attitude compared to those with a master’s degree. The odds of having a positive attitude toward Mpox prevention decrease with an increase in the monthly income of the health professional. The healthcare professionals with a monthly income in the range of 8,018–9,057 ETB were approximately three times (AOR = 2.83, 95% CI = 1.51, 5.31) more likely to have a positive attitude when compared to those with a monthly income of more than 15,000 ETB. Regarding religion, orthodox healthcare professionals were 1.6 times (AOR = 1.65, 95% CI = 1.01, 2.74) more likely to have a positive attitude when compared to their Muslim counterparts (Table 5).

**Table 5:** Multivariable logistic regression model for the attitude toward Mpox infection prevention and associated factors among frontline healthcare professionals in Ethiopia, 2024.

Variables	Categories	COR	AOR	*p*-value
Age category in years	15–24	Ref	Ref	
	25–30	1.33 (0.68, 2.63)	1.27 (0.61, 2.64)	0.522
	31–34	1.16 (0.60, 2.23)	1.15 (0.55, 2.42)	0.698
	35–40	1.05 (0.50, 2.22)	1.10 (0.48, 2.52)	0.812
	>40	0.96 (0.40, 2.27)	1.00 (0.38, 2.60)	0.995
Sex of participants	Female	Ref	ref	
	Male	1.75 (1.16, 2.65)	2.07 (1.30, 3.29)	0.002*
Educational status	Master’s	Ref	ref	
	Degree	1.17 (0.86, 1.59)	0.91 (0.68, 1.41)	0.921
	Diploma	2.19 (1.33, 3.62)	1.96 (1.04, 3.68)	0.036*
Monthly income in ETB	600–6,193	2.34 (1.35, 4.06)	2.19 (1.15, 4.13)	0.016*
	6,194–8,017	1.93 (1.14, 3.29)	2.10 (1.18, 3.70)	0.011*
	8,018–9,057	2.18 (1.54, 5.10)	2.83 (1.51, 5.31)	0.001*
	9,058–15,390	1.15 (0.68, 1.96)	1.19 (0.69, 2.06)	0.522
	>15,390	Ref	Ref	
Religion	Muslim	Ref	Ref	
	Orthodox	1.54 (0.95, 2.49)	1.65 (1.01, 2.74)	0.048*
	Protestant	1.32 (0.82, 2.10)	1.29 (0.85, 2.27)	0.184
	Others	1.37 (0.69, 2.69)	1.48 (0.73, 3.02)	0.271
Residence	Urban	Ref	Ref	
	Rural	0.96 (0.59, 1.55)	0.87 (0.52, 1.44)	0.599
COVID-19 vaccination history	No	Ref	Ref	
	Yes	1.16 (0.84, 1.59)	0.97 (0.69, 1.37)	0.188
Knowledge	Poor	Ref	Ref	
	Good	1.49 (1.11, 2.00)	1.41 (1.02, 1.91)	0.033*

COR, crude odds ratio; AOR, adjusted odds ratio; ref, reference category; ETB, Ethiopian Birr. *denotes a significant *p*-value < 0.05.

## Discussion

In this study, participants in the age group of 25–30 years were found to have higher knowledge of Mpox and its prevention. A comparable finding was reported from Cameroon (11). The possible explanation is that this age group is a part of the social media-friendly group and has improved internet access, which could result in a higher likelihood of acquiring Mpox information (11, 25). Public health experts need to address the driving factors of the age difference in knowledge by leveraging the available information.

This study reveals that males were more likely to possess higher knowledge about the Mpox infection. This finding contradicts studies of medical students in 27 countries (26, 29) and Northwest China (24, 27), where male healthcare workers showed less positive attitudes toward Mpox than females. Conversely, studies from other Arab nations reported no significant gender-based differences in Mpox knowledge (28). These discrepancies may be attributed to variations in study populations, cultural differences, or recent changes in public health awareness campaigns that target specific demographics. The conflicting findings underscore the necessity for further studies to explore the underlying factors contributing to these variations. Addressing this knowledge gap through ongoing healthcare provider training, targeted health education campaigns, and gender-specific interventions is crucial to enhancing public understanding of Mpox infection.

Vaccination against COVID-19 was also found to increase the knowledge of Mpox among healthcare professionals in the country. This is in line with previous studies conducted among Ethiopian healthcare professionals (23) and the general population of Nepal (29). This could be due to the common understanding that COVID-19 has provided healthcare professionals with an extensive range of knowledge that can be utilized to help prevent the spread of Mpox. Furthermore, people who received the COVID-19 vaccine may be more prepared to stay informed about Mpox (23, 30).

In our study, male participants were more likely to have a positive attitude than their female counterparts. These findings conflict with the studies that examined the knowledge and attitude about Mpox of medical students in 27 countries of the three continents (31), and China (north-west China) (25), in which male healthcare professionals were less likely to exhibit a positive attitude toward Mpox compared to females. The reason for this difference could depend on the extent to which women are educated in each country, as the authors of the previous study also speculated. The current finding implies that health education and training to improve healthcare workers’ attitudes toward Mpox infection should target female healthcare providers in Ethiopia.

In this study, the odds of having a positive attitude toward Mpox and its prevention were approximately two times higher among healthcare workers who had a diploma compared to those who had a master’s degree. However, other studies conducted in Lebanon (32), Kuwait (33), Jordan (34), and Malaysia (35) revealed that a higher level of education was directly related to a more positive attitude toward the disease. This difference may stem from seniority and education level potentially correlating with greater outbreak management knowledge and attitude. This result highlights the need for providing training and educational campaigns for healthcare workers with higher educational qualifications in Ethiopia.

Consistent with studies from Bangladesh (36), Lebanon (32), Jeddah (37), Turkey (38), and China (39), healthcare workers with good Mpox knowledge showed significantly more positive attitudes toward prevention than others. In our study, the healthcare workers who had good knowledge about Mpox infection exhibited significantly higher positive attitudes toward the disease and its prevention than their counterparts. The current finding is also supported by a study conducted in Northwest Ethiopia (23), which revealed that having access to information about Mpox is more likely associated with positive attitudes among healthcare workers. This may be because an increased level of knowledge regarding an emerging infection can increase confidence in the management and diagnosis of the disease. This observation emphasizes that enhancing the knowledge of healthcare professionals should be considered in interventions aiming to improve attitudes and overall preparedness for an outbreak.

## Strengths and limitations

### Strengths

Mpox has been declared a public health emergency of both global and continental significance, highlighting the urgent need for comprehensive studies. In this regard, conducting a nationwide study with such a large sample size can produce a reliable estimate. Therefore, this study addresses the gap in knowledge regarding the Mpox outbreak in Ethiopia at the national level, providing valuable insights. The findings will assist policymakers and clinicians in developing targeted intervention strategies to effectively combat the outbreak.

## Limitations

Cautiousness is very crucial when generalizing these findings to healthcare professionals in Ethiopia. As the survey was conducted online, professionals in regions with limited or no internet access may not have been included, potentially affecting the generalizability of the estimate to rural areas. As a result, the findings may not fully capture perspectives from areas with internet connectivity problems.

Furthermore, this study is also subject to self-report bias, which can affect the accuracy of responses. Participants may have provided socially desirable answers rather than their true perspectives, leading to a potential overestimation of the knowledge and attitude level of healthcare professionals toward Mpox and its prevention. Given these factors, the responses may not fully reflect the participants’ actual views, and caution is advised when interpreting the findings.

## Conclusion and recommendations

In Ethiopia, older adults and female frontline healthcare professionals exhibited lower knowledge about the Mpox infection. Conversely, higher knowledge was observed among frontline healthcare professionals who had been vaccinated against COVID-19. Hence, from a policy perspective, targeting these specific groups for further educational interventions can be associated with improved control and prevention of Mpox outbreaks. Further studies should prioritize elucidating additional factors associated with frontline healthcare professionals’ knowledge and attitudes toward Mpox infection.

## Data Availability

The raw data supporting the conclusions of this article will be made available by the authors, without undue reservation.
